# Supplementary material for: Mediation Analysis of Waist Circumference in the Association of Gut Microbiota with Insulin Resistance in Children
Source: Children (Basel). 2023 Aug 14;10(8):1382. doi: 10.3390/children10081382 (PMC10453241; doi:10.3390/children10081382)
Supplement: Supplementary file 1 [file children-10-01382-s001.zip › Table S1-S2.pdf]

Table S1. Specific universal primers.

Target	Sequence 5′ to 3′	Tm °C	References
A. muciniphila	F: CAGCACGTGAAGGTGGGGAC<br>R: CCTTGCGGTTGGCTTCAGAT	63	51
L. casei	F: CTATAAGTAAGCTTTGATCCGGAGATTT<br>R: CTTCCTGCGGGTACTGAGATGT	59	
L. paracasei	F: ACATCAGTGTATTGCTTGTCAGTGAATAC<br>R: CCTGCGGGTACTGAGATGTTTC	60	52
L. reureti	F: ACCGAGAACACCGCGTTATTT<br>R: CATAACTTAACCTAAACAATCAAAGATTGTCT	59	
S. aureus	F: GCC ACG TCC ATA TTT ATC AGT TC<br>R: GAT ACA CCT GAA ACA AAG CAT CC	60	Designed by INTEGRATED DNA TECHNOLOGIES ™
Universal	F: AAACTCAAAKGAATTGACGG<br>R: CTCACRRCACGAGCTGAC	58	53

F: Forward; R: Reverse; Tm: Melting temperature.

Table S2. Metabolic status of participants according to BMI Z-scores.

Characteristics[a] n=533	Normal weight n=265 (51%)	Overweight/Obesity n=268 (49%)	*p*-value
**Metabolic Status**			
Glucose (mg/dL)	82 (75-88)	83 (78-88)	0.189
Cholesterol total (mg/dL)	158 (137-177)	165 (145-189)	**0.004**
Triglycerides (mg/dL)	68 (53-88)	101 (73-146)	**<0.001**
LDL (mg/dL)	98 (84-115)	108 (93-127)	**<0.001**
HDL (mg/dL)	54 (47-64)	48 (39-58)	**<0.001**
Insulin (mU/L)	1.9 (1.5-2.7)	4.0 (2.0-7.5)	**<0.001**
Adiponectin (μg/mL)	5.67 (4.95-6.53)	4.74 (4.14-5.69)	**<0.001**

[a] Values represented the median (p25 and p75) or percentages. Mann- Whitney U test. LDL: low-density protein; HDL: high-density protein. Statistically significant differences are marked in bold p <0.05.
