# Supplementary figures and images for: Mediation Analysis of Waist Circumference in the Association of Gut Microbiota with Insulin Resistance in Children
Source: Children (Basel). 2023 Aug 14;10(8):1382. doi: 10.3390/children10081382 (PMC10453241; doi:10.3390/children10081382)

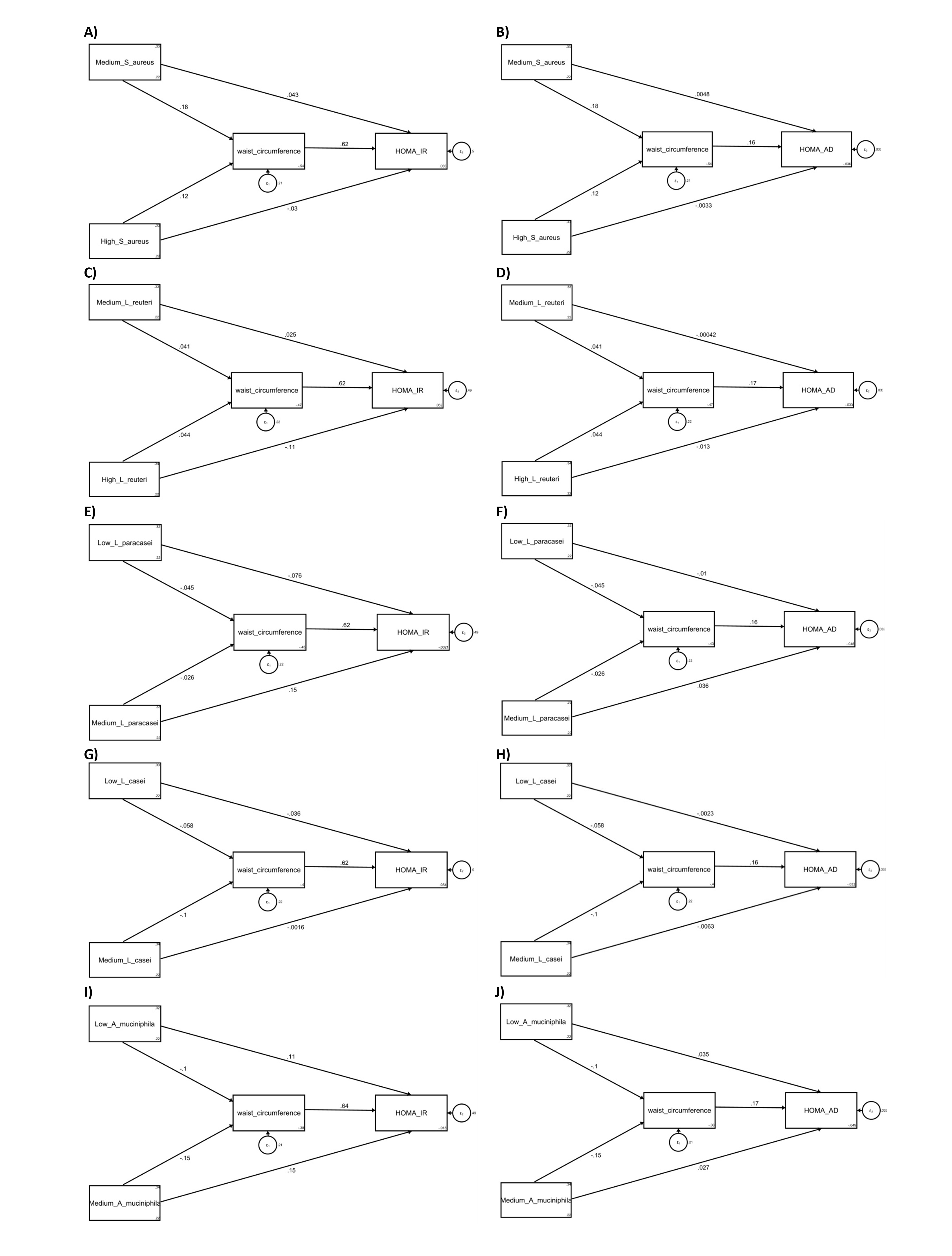

Supplement: Supplementary file 1 [file children-10-01382-s001.zip › Fig S1 Path Analysis.jpg]

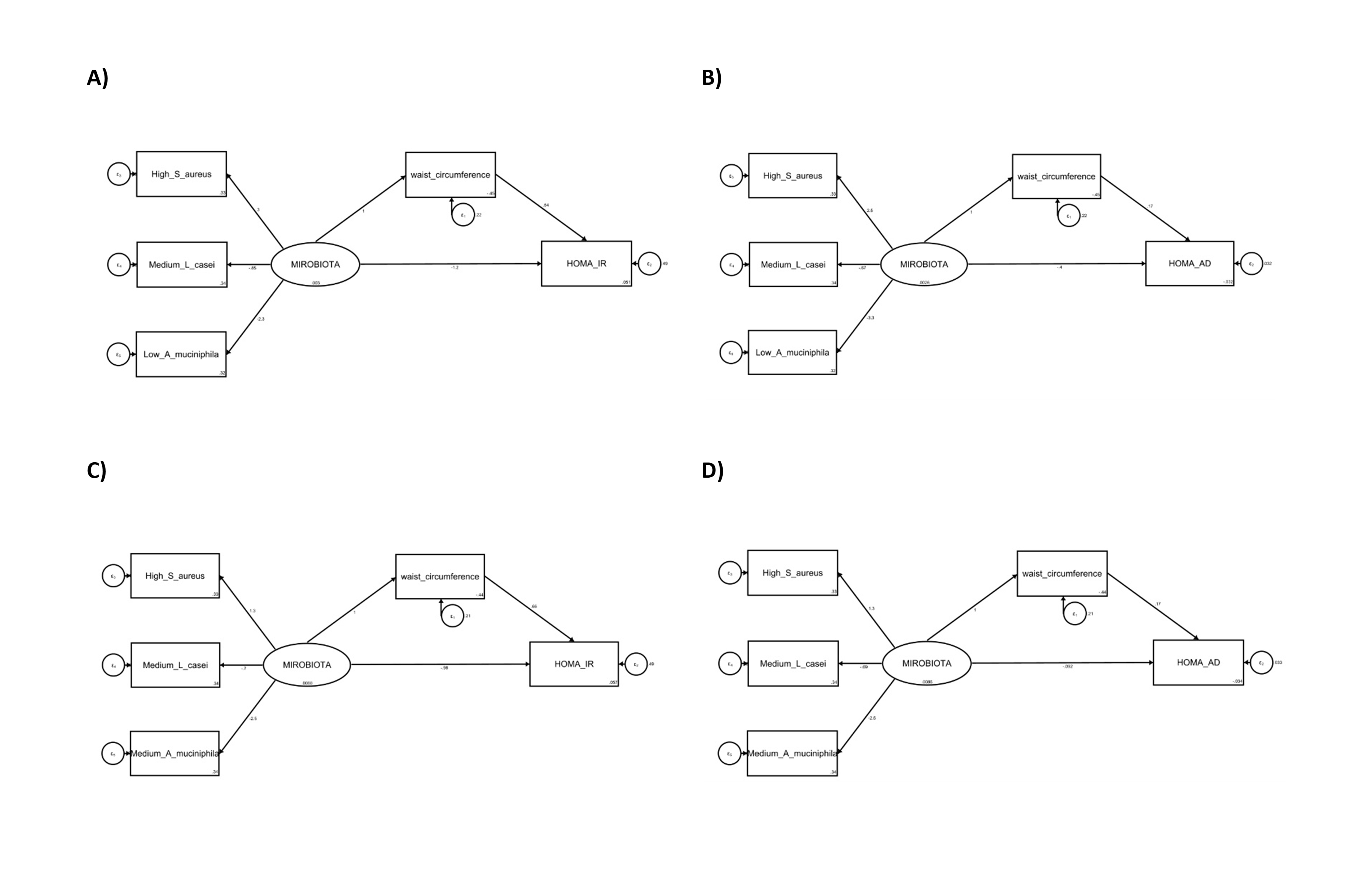

Supplement: Supplementary file 1 [file children-10-01382-s001.zip › Fig S2 Structural Equation Modeling.jpg]
